# F57. CORRELATION FACTORS OF ABNORMAL MENSES IN SCHIZOPHRENIA TREATMENT WITH RISPERIDONE

**DOI:** 10.1093/schbul/sby017.588

**Published:** 2018-04-01

**Authors:** Fang Dong, Qijing Bo, Chuanyue Wang

**Affiliations:** Beijing Anding Hospital of Capital Medical University

## Abstract

**Background:**

A significant percentage of women taking antipsychotic medication may be suffering from abnormal menses during their treatment, which influences both fertility and adherence to medication. It is particularly common in patients prescripted with risperidone. This study aimed to identify the risk factors for abnormal menses in female individuals with schizophrenia during risperidone treatment, especially the relationship between abnormal menses and the dose or the length of the medicine.

**Methods:**

This study used a retrospective data. 202 female patients diagnosed with schizophrenia using risperidone were screened. Doses and length of treatment with risperidone were various. 38 were excluded for their menstrual irregularities before treatment, in which 4 amenorrhea and 15 menopause. 164 female patients included, but 3 of them absent of data. 161 female patients included in analyses at last.

**Results:**

Of the 161 patients, 119 were eumenorrhea up to our analyses, and other 42 abnormal menses, including 23 menstrual irregularities, 8 amenorrhea and 11 oligomenorrhea. There was no statistical difference in age (32.0 ± 8.6 vs. 31.4 ± 10.1) (years), education (12.2 ± 2.3 vs. 12.6 ± 2.2) (years), age at onset 26.7 ± 8.0 vs. 24.8 ± 8.4) (years), duration of illness (5.8 ± 5.2 vs. 7.0 ± 7.7) (years), PANSS total score (37.2 ± 8.8 vs. 38.1 ± 7.0) between normal group and abnormal group. There was also no statistical difference in risperidone dose at baseline (4.3 ± 0.7 vs. 4.3 ± 0.5) (mg/d), total treatment in this episode (5.3 ± 4.7 vs. 5.4 ± 5.4) (months), overall length of risperidone treatment in this episode (86.7 ± 62.0 vs. 98.6 ± 73.5) (days), length of risperidone treatment at optimal therapeutic dose (63.0 ± 64.5 vs. 51.3 ± 26.7) (days).

**Discussion:**

Some research suggests antipsychotic-induced abnormal menses is related to medication-induced high prolactinemia level and low estradiol level pretreatment. But few study reports the relationship between abnormal menses and the dose or the length of the medicine. This study got negative results, which suggest the occurrence of abnormal menses widely depend on individual quality rather than the length and the dose of the antipsychotic. But there are some limits in the study. First, the dosage range among these subjects were relatively narrow. And then, the length of risperidone treatment is generally short. In the next step of research, we will improve these two points.

